# Training and Toolkit Resources to Support Implementation of a Community Pharmacy Fall Prevention Service

**DOI:** 10.3390/pharmacy7030113

**Published:** 2019-08-09

**Authors:** Jessica M. Robinson, Chelsea P. Renfro, Sarah J. Shockley, Susan J. Blalock, Alicia K. Watkins, Stefanie P. Ferreri

**Affiliations:** 1 UNC Eshelman School of Pharmacy, University of North Carolina at Chapel Hill, Chapel Hill, NC 27599, USA; 2 Department of Clinical Pharmacy and Translational Science, University of Tennessee Health Science Center College of Pharmacy, Memphis, TN 38163, USA; 3 Durham VA Health Care System, Durham, NC 27705, USA

**Keywords:** community pharmacy services, medication therapy management, implementation science, capacity building, staff development, fall prevention, aged

## Abstract

Community pharmacies are an ideal setting to manage high-risk medications and screen older adults at risk for falls. Appropriate training and resources are needed to successfully implement services in this setting. The purpose of this paper is to identify the key training, tools, and resources to support implementation of fall prevention services. The service was implemented in a network of community pharmacies located in North Carolina. Pharmacies were provided with onboard and longitudinal training, and a project coach. A toolkit contained resources to collect medication information, identify high-risk medications, develop and share recommendations with prescribers, market the service, and educate patients. Project champions at each pharmacy received a nine-question, web-based survey (Qualtrics) to identify usefulness of the training and resources. The quantitative data were analyzed using descriptive statistics. Thirty-one community pharmacies implemented the service. Twenty-three project champions (74%) completed the post-intervention survey. Comprehensive onboard training was rated as more useful than longitudinal training. Resources to identify high-risk medications, develop recommendations, and share recommendations with prescribers were considered most useful. By providing appropriate training and resources to support fall prevention services, community pharmacists can improve patient care as part of their routine workflow.

## 1. Introduction

Falls are the leading cause of fatal and non-fatal injury for older adults, causing more than seven million injuries annually in the United States [1]. Each year, more than one-quarter of older adults will experience a fall, which can lead to decreased quality of life due to functional decline [2]. In addition, the financial burden to individuals, families, and the overall health care system cannot be overlooked—the total annual direct cost of fall-related medical care was estimated to be (US)$50 billion in 2015 [3]. In 2016, the U.S. Surgeon General, in partnership with the National Prevention Council, released the Healthy Aging in Action National Prevention Strategy to address national priorities [4]. Understandably, the expansion of effective fall prevention services was a key recommendation identified.

A variety of factors can lead to an increased risk of falls in older adults, including the use of medications [5]. This includes high-risk medication use (primarily medications with sedative/hypnotic effects), as well as polypharmacy (use of ≥4 chronic medications) [5,6]. In order to address the impact of falls on older adults, the Centers for Disease Control and Prevention (CDC) developed the Stopping Elderly Accidents, Deaths, and Injuries (STEADI) initiative [7]. STEADI offers resources for health care providers to assess and minimize fall-related risk factors such as medications linked to falls, home hazards, sensory impairment, and chronic conditions [8]. While originally developed for use in primary care, engagement has remained low in this setting [9,10]. With rising prescription medication use, polypharmacy, and patients receiving care from numerous providers [11,12], there is a distinct need for the expertise of a pharmacist to help manage medication-related fall risk. Due to their access to patients and medication records [13], community pharmacists are ideally positioned to partner with providers, payers, and public health stakeholders to reduce the risk of falls through screening, medication management, and referral.

Prior research indicates that community pharmacists successfully screened and made medication recommendations for older adults at risk for falls in a single community pharmacy [14,15,16]. In order to successfully integrate fall prevention clinical activities into multiple community pharmacies, several key factors must be addressed. Community pharmacists have indicated a variety of challenges associated with implementing new clinical services, including limited time for staff training, administrative burden, and lack of workflow integration [17,18]. These challenges can be addressed by providing appropriate implementation support. One such framework for supporting adoption and implementation of a new service is the Evidence-Based System for Innovation Support (EBSIS) model [19]. Key factors addressed by the EBSIS include the proactive use of tools, training, technical assistance, quality assurance, and quality improvement to build an organization’s capacity to implement innovations and achieve outcomes [19].

The purpose of this paper is to identify the key training, tools, and resources to support implementation of fall prevention services within the community pharmacy setting. By providing appropriate support for new services, community pharmacists can improve patient care as part of their routine workflow.

## 2. Materials and Methods

The fall prevention service was developed using key concepts from the CDC STEADI initiative. The EBSIS framework was used to guide development of training, tools, and resources, as well as technical support and quality improvement. A project coach was deployed from the investigative team to ensure fidelity of training among pharmacy staff and to provide technical support and feedback. The service was piloted over a nine-month period, from October 2017 to June 2018. The Office of Human Research Ethics, Institutional Review Board (IRB) at the University of North Carolina at Chapel Hill (UNC) approved this study (#16-2619).

### 2.1. Service Description

**Figure 1:**
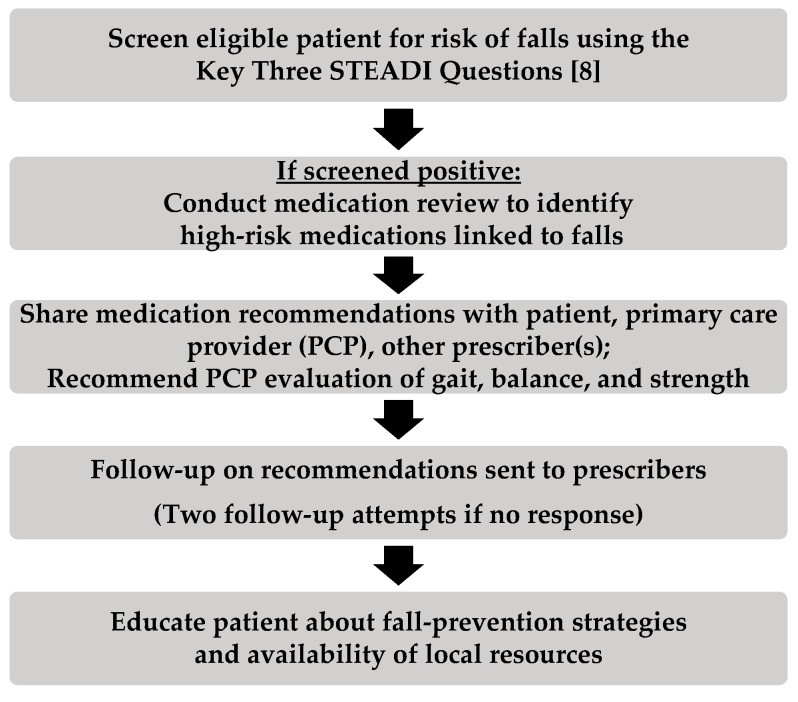
Community pharmacy fall prevention process.

The fall prevention service used in community pharmacies involved screening patients at risk for falls, assessing modifiable risk factors (i.e., high-risk medications), sharing recommendations for safer alternatives with prescribers, and educating the patient. The fall prevention service process is shown in Figure 1. Patients were eligible for screening if they were ≥65 years of age and took ≥four chronic medications or at least one high-risk medication.

### 2.2. Participants

The service was implemented in community pharmacies located within a North Carolina community pharmacy enhanced services network (NC CPESN^®^) who were participating in the intervention arm of a randomized-controlled trial. A project champion was identified by management from each pharmacy to lead the service. The project champion was responsible for staff training, as well as monitoring of service implementation and documentation. The individual could be a pharmacist or technician. At the conclusion of the study, project champions were responsible for completing a post-intervention survey on behalf of the pharmacy.

### 2.3. Tool Development

EBSIS defines tools as resources to “organize, summarize, and/or communicate knowledge” and recommends soliciting end-user feedback to develop high-quality and well-designed tools in order to achieve targeted outcomes [19]. Study investigators developed a 66-page toolkit to support implementation of the fall-prevention service. In addition to containing key information about older adult falls, fall-prevention strategies, and service protocols, the toolkit contained resources to facilitate effective patient intervention and care coordination (Table 1). Resources were designed to be easily integrated into pharmacy workflow and emphasized using non-clinical staff (technicians) to support the service. This included tools to support technician-driven patient screening and collection of relevant medication information prior to a pharmacist-provided medication review. In order to optimize the pharmacist medication review and recommendation process, tools were provided to assist with clinical decision-making and interdisciplinary communication. Patient education materials, including STEADI patient education brochures [20,21,22], were provided for referring patients to evidence-based fall prevention strategies outside the scope of pharmacy practice. In order to enhance the usability of tools for internal and external use, study investigators sought feedback from an advisory panel of pharmacists, providers, and provider office staff. In addition, feedback was solicited from project champions to make improvements to tools and resources throughout implementation. A detailed outline of toolkit contents is provided in the Supplementary Materials, along with individual tools developed.

### 2.4. Training Approach

A multi-faceted approach was developed to ensure that pharmacy staff received training that aligned with their roles and responsibilities in this project. According to the EBSIS framework, training should include “planned, instructional activity intended to facilitate the acquisition of knowledge, skills and attitudes in order to enhance learner performance [19].” Study investigators utilized a combination of both onboard and longitudinal training to accomplish training objectives. Onboard training took place prior to the new service being implemented and it was comprised of both required and optional training opportunities. For onboard training, project champions were required to (1) participate in a one-hour, live webinar, (2) review toolkit resources, and (3) participate in a 45- to 60-minute pharmacy site visit. To provide project champions with adequate opportunity for webinar participation, the webinar was held on three separate occasions during the work week to accommodate conflicting work schedules. All staff were encouraged to attend live onboard training with the project champion. The webinar was also recorded and made available online to accommodate staff unable to join live. Optional onboard training included STEADI: The Pharmacist’s Role in Older Adult Fall Prevention, a free online continuing pharmacy education (CPE) program developed by the American Pharmacists Association (APhA) and CDC [24]. A second optional onboard training was Collaborative Approach to Falls Assessment and Prevention, a one-day live workshop offered to attendees at an annual state pharmacy association meeting [25]. This training provided foundational training on the pathophysiologic and pharmacologic basis for falls in older adults. Study investigators were not associated with the workshop.

Longitudinal training consisted of six “Quick Tips” webinars held between October 2017 and February 2018 (5 months). These 30-minute webinars provided time for the research team to answer pharmacy staff questions and present best practices for successful patient interventions. Topics covered workflow and staff integration, patient and prescriber engagement, and examples of programmatic success from participating pharmacies. Speakers included pharmacists and physicians from the research team, as well as speakers from participating pharmacies. Pharmacy staff received “Quick Tips” emails on a bi-weekly to monthly basis from between October 2017 and June 2018 (9 months). The emails contained brief strategies to improve patient and provider engagement, as well as addressing study-related project management topics. An outline of training is provided in the Supplementary Materials. All training was provided to pharmacy staff free of charge, with the exception of the one-day live workshop, which required a conference registration fee and travel.

To minimize the impact of staff turnover on service implementation fidelity, as well as to facilitate training of new staff, training materials were recorded and provided to project champions. This included recording all webinars and offering print copies of webinar slides. The one-day live workshop and site visit were exceptions, as it was not possible to capture the content provided in these training sessions. The online continuing education program was available through the duration of the study.

### 2.5. Technical Support and Quality Assurance/Quality Improvement

A project coach was responsible for technical support and quality assurance/quality improvement, modeling activities after the EBSIS framework. EBSIS defines technical support as “individualized, hands-on approach to building an entity’s capacity for quality implementation of innovations” and quality assurance/quality improvement” [19]. The project coach managed longitudinal training, provided feedback on quality of documentation, and offered general support. Utilizing the Plan-Do-Study-Act (PDSA) cycle [26], the coach assessed service implementation and fidelity for each pharmacy on a monthly basis. Areas of challenge were subsequently addressed in webinars and emails. In addition, pharmacy staff had access to the coach for one-on-one training by phone, email, or during the on-site visit. The project coach collected information about successes and challenges reported during these interactions. Pharmacies were provided with a “report card” to compare their progress with patient screening to other pharmacies participating in the pilot. This concept was adapted from a study by Shultz et al., that used normative social influence to encourage residents to reduce power consumption by comparing their power utilization with neighbors [27]. Investigators for this study employed the concept to encourage pharmacy staff to increase patient screening rates. The report anonymously compared the pharmacies’ proportion of patients screened and described whether the pharmacy was performing at, above, or below the average screening rate. Report cards were accompanied by suggestions to improve screening rates. Report cards were shared a total of two times during the screening period, which occurred between October 2017 and January 2018.

### 2.6. Program Assessment

Investigators documented project champion participation in the required and optional onboard training events during the site visit. At the conclusion of the study, project champions were asked to complete a nine-question web-based survey (Qualtrics, Provo, UT, USA) about their participation in both onboard and longitudinal training, as well as to rate the usefulness of the tools and training resources provided. The survey was developed through a review process with the investigative team to ensure effective collection of relevant data. Reminder emails were sent three times—one, two, and three weeks after the survey was initially sent. The quantitative data were analyzed using descriptive statistics. The nine-question survey is available in the Supplementary Materials.

## 3. Results

### 3.1. Baseline Characteristics

Thirty-one independent community pharmacies implemented the service. Seven pharmacies (23%) were single, independently-owned stores, while the remaining 24 pharmacies (77%) were affiliated with independently-owned chains (≥three locations). Pharmacists (owner or staff) served as project champions in 30 pharmacies (97%), while one technician (3%) served in the role. Of these, 23 (74%) completed the post-intervention survey. The majority (65%) of project champions had practiced in their respective pharmacy for more than five years. See Table 2.

### 3.2. Usefulness of Toolkit Resources

Project champions rated toolkit resources on a Likert scale of 1–3 (1 = not useful, 2 = somewhat useful, 3 = very useful) as shown in Table 3. The high-risk medication index and high-risk medication algorithms were rated most useful.

### 3.3. Participation and Usefulness of Training

Twenty-eight pharmacies (90%) participated in all three required onboard trainings. Four pharmacies (13%) participated in the required onboard training plus an optional training program. Three pharmacies (10%) attended ≤two trainings. In the first six months of the study, the project coach logged 109 encounters with pharmacies, spending approximately 27 hours providing coaching, feedback, and toolkit customizations for pharmacies. In the final three months of the study, no formal coaching occurred, and interactions were minimal, as most pharmacies had fully implemented the service by that point. Twenty-three survey participants rated the usefulness of training and training topics. On a Likert scale of 1–3 (1 = not useful, 2 = somewhat useful, 3 = very useful) as shown in Table 4, the onboard training webinar, onboard site visit, and optional live workshop were most highly rated as very useful.

### 3.4. Toolkit Resource Changes

During the first six months of the study, the project coach asked for suggestions to improve the usability of study toolkit resources. Most suggestions were related to forms that would be shared externally with providers. Approximately half of pharmacies requested that forms be provided in an electronic fillable PDF format for better integration into their workflow and pharmacy management software. For pharmacies that preferred to use hardcopies, they recommended that forms be supplied as single-sided instead of double-sided, since many fax machines have single-sided feeds. In addition, the project coach noted that the original in-color provider communication forms did not appear properly after faxing due to an issue with the design. The forms were redesigned in black and white to enhance contrast and improve the readability of communications sent to providers. Lastly, 15 pharmacies (48%) requested to have forms customized to include their pharmacy logo in order to enhance pharmacy brand recognition.

### 3.5. Program Successes and Challenges

The project coach identified a variety of successes and challenges related to implementation of the fall-prevention service. In addition to technical challenges related to toolkit resources as mentioned in the previous section, the project coach recounted that implementation of the service was delayed, paused, or completely stopped due to staff turnover in three participating pharmacies. Successes reported by pharmacy staff included sharing with the project coach that workflow design and tools facilitated participation by all staff, including technicians and students. Staff reported that the toolkit was helpful because it contained all the resources needed to effectively implement the fall-prevention service and that clinical decision-making tools helped them make recommendations in a more timely and efficient manner. Lastly, pharmacy staff reported that having access to a project coach to help troubleshoot toolkit resources or workflow issues helped them implement changes faster than if they had been responsible for identifying solutions themselves.

## 4. Discussion

This study used the EBSIS implementation framework to guide development of training and toolkit resources, as well as to guide use of technical support and quality assurance/quality improvement for a fall-prevention service within the community pharmacy setting. While each component of the framework can be used independently, they work in tandem to help organizations avoid common implementation challenges [19]. According to the framework, each component should enhance the previous component, as well as facilitate use of the next component. For example, development of tools precedes development of training, which precedes development of technical support, and so on. By using this framework, study investigators were able to anticipate the implementation needs of pharmacies and proactively offer solutions to common challenges. This study emphasized developing tools and resources to help reduce workflow barriers experienced by pharmacy staff. For example, tools related to making clinical decisions (i.e., high-risk medication index and algorithms) are a valuable resource to improve pharmacist efficiency when developing recommendations. Another challenge experienced in this setting is communicating information externally to other members of the health care team. When developing a new service, consideration should be given to developing tools that optimize communication. Communication tools should anticipate user needs, be easy to read and understand, and provide valuable information. The end-user experience should be accounted for by soliciting feedback during tool development from those sharing and receiving the information. In this project, we solicited feedback from pharmacists, providers, and provider office staff to help improve the quality and usability of tools.

Previous studies have identified lack of time and training as a barrier for implementing new community pharmacy services [28,29,30,31], thus emphasizing the need to leverage onboard training opportunities. This approach is confirmed by the Core Implementation Components Framework, which recommends pre-intervention training as an essential step to provide staff with appropriate background information, teach new processes, and provide feedback prior to implementation [32]. Essential clinical training, service protocols, and implementation training should occur at program initiation in order to maximize staff participation and buy-in, and to increase likelihood of implementation fidelity at launch. While the EBSIS framework does not provide direct guidance on the type or dose of training to be delivered at onboard versus longitudinal training, it emphasizes careful planning and development of training prior to implementation [19]. In this study, comprehensive onboard training focused on workflow and clinical processes, providing pharmacy staff with instruction for implementing the fall prevention service. Conversely, longitudinal training was often focused more narrowly on specific challenges in the pharmacies. When surveyed, project champions tended to rate onboard training as more useful than longitudinal training. In addition, the EBSIS framework recommends that training be conducted in a group session [19]. Due to geographical challenges, it was not possible to provide traditional group training among multiple pharmacies. To overcome this challenge, study investigators used live webinars as an alternative. Through this medium, pharmacy staff had adequate opportunity to verbalize questions, address challenges, and problem-solve. In addition, consideration should be given to the timing of live onboard training. A final recommendation is to vary the schedule of live trainings offered. In this study, both onboard and longitudinal trainings were offered at varying times of day, when possible, to allow participants to work flexibly around peak pharmacy hours and staffing schedules.

Success of a new clinical service is often dependent on participation from several staff members within a pharmacy and can be affected by staff turnover. The impact of staff turnover on general organizational performance is well-established in the literature [33], but investigators were unable to identify research that studied the impact of pharmacy staff turnover on delivery of clinical pharmacy services. Absent supporting data, we encouraged participating pharmacies to involve either all or multiple staff in the service, thus hopefully decreasing the impact of staff turnover. This project provided all training materials in print and/or recorded format to ensure that new staff were adequately prepared to deliver the service following turnover.

This study utilized a project coach from the research team to provide longitudinal coaching and feedback on implementation of the fall prevention service. The number of project coach encounters in the first six months suggests the need for a point person to manage implementation needs. Implementation changes within the first few weeks of the study were often related to workflow integration and customization of toolkit resources to leverage pharmacy branding. Pharmacy staff reported that having a highly-responsive individual to handle these needs helped them integrate the service more quickly and efficiently. Due to the limited feasibility of a project coach in most practice-based settings, the functions of this role should be incorporated into that of a project champion or clinical services manager in a larger organization. These functions include ensuring that staff receive adequate onboard training; providing access to tools and resources; making improvements to tools, resources, and workflow processes; monitoring quality of service implementation and documentation; and providing feedback.

### Limitations

Pharmacies participating in this study were independent pharmacies in an established community pharmacy enhanced services network, limiting generalizability to a broader audience of community pharmacies. Pharmacies in this network have experience with integrating new services into workflow and may have had designated clinical staff to implement the service. Additionally, due to its small sample size, it is difficult to generalize results.

## 5. Conclusions

Community pharmacists are a resource for identifying and assessing fall-related risk factors in older adults. This study identified the training and toolkit resources used to support implementation of a fall prevention service within a community pharmacy. The training, tools, and support allowed community pharmacists and staff to integrate the service into existing workflow processes. Future research should build on these findings and assess the direct impact of training on success of service implementation and patient outcomes. By providing appropriate training and resources to community pharmacy staff support fall prevention services, they can improve patient care as part of their routine workflow.

## Figures and Tables

**Table 1:** Community pharmacy fall prevention toolkit resources.

Tool	Function
High-risk medication algorithms (nine total)	Identify drug class-specific risk factors and alternative treatment strategies
High-risk medication index	Identify high-risk medications and correlating drug class
Prescriber communication form [23]	Transmit patient screening results and pharmacists’ recommendations to prescriber and/or primary care provider
Cover fax form	Prompt other health care providers to recall purpose of community pharmacy fall prevention service
Prescriber response form [23]	Gather prescriber acceptance/rejection of pharmacists’ recommendations
Medication checklist	Collect list of high-risk medications and relevant health information
Prescriber flyer	Promote community pharmacy fall prevention service to prescribers and/or primary care provider
Patient education brochures ^†^	Educate patients about risk factors and evidence-based strategies to prevent falls
Community resources	Identify local/regional evidence-based fall prevention resources for patient referral

† Check for Safety [20], What You Can Do to Prevent Falls [21], and Postural Hypotension [22]; provided in English and Spanish.

**Table 2:** Project champion baseline characteristics.

Characteristic	*N* (%)
**Role** (*N* = 31)	
Technician	1 (3)
Owner Pharmacist	6 (19)
Staff Pharmacist	24 (77)
**Education and Training** (*N* = 23) ^†,‡^	
Technician Certification (CPhT)	1 (4)
Board Certification	2 (9)
Clinical Pharmacy Practitioner	2 (9)
Residency	3 (13)
Pharmacist graduate (BS Pharm)	8 (35)
Pharmacist graduate (PharmD)	14 (61)
**Years Worked at Site** (*N* = 23) ^†^	
<1 year	1 (4)
1–5 years	7 (30)
>5 years	15 (65)

† Of the 31 project champions, 23 responded to the post-intervention survey. ‡ Respondents could make multiple selections for education and certifications. Therefore, percent total does not add to 100.

**Table 3:** Reported usefulness of toolkit resources.

Resources	Mean (SD) ^†^
High-risk medication algorithms (*N* = 22) *	2.95 (SD = 0.21)
High-risk medication index (*N* = 22) *	2.91 (SD = 0.29)
Prescriber communication form (*N* = 22) *	2.91 (SD = 0.29)
Cover fax form (*N* = 22) *	2.77 (SD = 0.43)
Prescriber response form (*N* = 22) *	2.77 (SD = 0.43)
Medication review checklist (*N* = 18) *	2.67 (SD = 0.59)
Prescriber marketing flyer (*N* = 18) *	2.50 (SD = 0.62)
STEADI patient education brochures (*N* = 20) *	2.45 (SD = 0.60)
Community resources (*N* = 19) *	2.11 (SD = 0.66)

* *N* indicates the number of project champions who reported using the resource. † Project champions rated resources in which they had participated on a Likert scale where 1 = not useful, 2 = somewhat useful, and 3 = very useful.

**Table 4:** Reported usefulness of training activities.

Training Activities	Mean (SD) †
Live workshop (*N* = 3) *^,^^‡^	3.00 (SD = 0)
Onboard training webinar (*N* = 22) *	2.86 (SD = 0.35)
Site visit (*N* = 21) *	2.71 (SD = 0.56)
Coaching (*N* = 17) *	2.59 (SD = 0.51)
Quick tips webinars (*N* = 17) *	2.35 (SD = 0.61)
Quick tips emails (*N* = 21) *	2.29 (SD = 0.56)
APhA Online CPE (*N* = 15) *	2.27 (SD = 0.59)
**Webinar Topics**	
Workflow integration (*N* = 17) *	2.41 (SD = 0.62)
Engaging with prescribers (*N* = 17) *	2.38 (SD = 0.62)
Engaging with patients (*N* = 16) *	2.35 (SD = 0.61)
Peer examples (*N* = 17) *	2.35 (SD = 0.49)

* *N* indicates the number of project champions who reported attendance. † Project champions rated training in which they had participated on a Likert scale where 1 = not useful, 2 = somewhat useful, and 3 = very useful. ‡ Referred to as “NCAP 2017 workshop” on survey.

## Supplementary Materials

The following are available online at https://www.mdpi.com/2226-4787/7/3/113/s1, Figure S1: Cover fax form, Figure S2: High-risk medication algorithms, Figure S3: Medication review checklist, Figure S4: Prescriber marketing flyer, Table S1: Implementation training and resources, Table S2: High-risk medication index, Table S3: Project champion survey. References [34,35] are cited in the Supplementary Materials.
