# Supplementary material for: Training and Toolkit Resources to Support Implementation of a Community Pharmacy Fall Prevention Service
Source: Pharmacy (Basel). 2019 Aug 9;7(3):113. doi: 10.3390/pharmacy7030113 (PMC6789452; doi:10.3390/pharmacy7030113)
Supplement: Supplementary file 1 [file pharmacy-07-00113-s001.pdf]

Table S1. Implementation training and resources.

STEP 1: ONBOARD TRAINING
**TOOLKIT REVIEW**
66-page toolkit mailed to pharmacies three weeks prior to live implementation. Pharmacy project champions were required to review toolkit prior to site visit.<br><br>Contents:<br>1. Checklist of training activities<br>2. Study overview<br>• Study objectives<br>• Introduction to research team members<br>• Glossary of key terms<br>• Project timeline<br>3. Fall prevention service<br>• Background information<br>• Process algorithm<br>• Stepwise processes for screening, medication review, sharing recommendations, patient education, and follow-up<br>4. Documentation requirements<br>5. Tools<br>• Cover fax form [*Figure S1*]<br>• High-risk medication algorithms [*Figure S2*]<br>• High-risk medication index [*Table S2*]<br>• Medication review checklist [*Figure S3*]<br>• Prescriber communication form [20]<br>• Prescriber marketing flyer [*Figure S4*]<br>• Prescriber response form [20]<br>6. Resources<br>• STEADI: The Pharmacist's Role in Older Adult Fall Prevention Resources List [34]<br>• Talking about Fall Prevention with Your Patients [35]<br>• North Carolina Community-based fall prevention resources [*Table S3*]<br>• STEADI patient education resources [21–23]<br>7. Acknowledgements
**LIVE WEBINAR**
A one-hour live webinar held on three alternating (morning/evening) dates, two weeks prior to live implementation. The webinar was recorded and disseminated to pharmacies to ensure training fidelity of future staff.<br>Topics:<br>1. Purpose of project<br>2. Fall-prevention processes<br>3. Documentation and compensation processes<br>4. Site visit expectations
**SITE VISIT**
A 45- to 60-minute site visit conducted by a member of the research team to meet with the pharmacy project champion and participating staff. Occurred during the first week of live implementation.<br><br>Topics:<br>1. Staff introductions<br>2. Housekeeping

- Obtain list of participating staff
- Update contact information
- Log training progress

3. Brief orientation
4. Practice case and review of toolkit resources
5. Q&A

OPTIONAL TRAINING

Two optional training opportunities:

- STEADI: The Pharmacist's Role in Older Adult Fall Prevention [24]
  - An online continuing pharmacy education module for pharmacists and technicians. Developed by the American Pharmacists Association (APhA) and the Centers for Disease Control and Prevention. Free registration for APhA members and non-members.
- Collaborative Approach to Falls Assessment and Prevention [25]
  - A one-day workshop held at North Carolina Association of Pharmacists Annual Meeting in September 2017. Provided comprehensive training on fall-risk assessment and prevention by an interdisciplinary team of pharmacists, an occupational therapist, and physical therapist. Free registration for meeting attendees.

STEP 2: LONGITUDINAL TRAINING

QUICK TIPS WEBINARS

Series of six 30-minute webinars held during the first six months of project. The webinars were recorded and disseminated to pharmacies to ensure training fidelity of future staff.

Topics

- Webinar 1: Review of screening and medication review processes
- Webinar 2: Peer example – implementing screening and medication review
- Webinar 3: How to talk about falls with your patients
- Webinar 4: Sharing fall risk information with prescribers
- Webinar 5: Peer example – collaborating with prescribers
- Webinar 6: Peer example – Incorporating STEADI into other pharmacy services

QUICK TIPS EMAILS

A biweekly-to-monthly email newsletter shared with pharmacy project champion and other pharmacy staff.

Topics:

- Best practices for:
  - Identifying and screening patients
  - Conducting medication reviews
  - Communicating with patients and prescribers
  - Documenting activities
  - Optimizing non-clinical staff
- Frequently asked questions
- Housekeeping
- News about project, falls research, state and national initiatives

STEP 3: PROJECT COACHING

A project coach was deployed from the investigative team to ensure fidelity of training among pharmacies and to provide technical support and feedback. The coach provided regular follow-up (i.e., every 1-2 weeks) by phone or email with pharmacy project champions for the first six months of the study. Follow-up continued to occur during the final three months of the project, but frequency was on an as-needed basis for each pharmacy.

Figure S1. Cover fax form.

STEADI
Stopping Elderly Accidents, Deaths & Injuries

UNC ESHELMAN SCHOOL OF PHARMACY

UNC SCHOOL OF MEDICINE
Division of Geriatric Medicine
Center for Aging & Health

Fax:

PRESCRIBER:	PHARMACIST:
FAX:	FAX:
PHONE:	PHONE:
SUBJECT: **FALLS-RISK ASSESSMENT**	DATE:
NO. PAGES:	

COMMENTS: **CONTAINS SENSITIVE PATIENT INFORMATION**

HELP US PREVENT FALLS IN YOUR OLDER ADULT PATIENTS

What does this mean for your practice?

- This is a pharmacy service supported by a grant from the Centers for Disease Control and Prevention to reduce the risk of falls in older adults through interprofessional collaboration.
- Through collaboration with your community pharmacy, you can meet your quality metrics (e.g. HEDIS measures, annual wellness visits)!
- You may receive a communication from a community pharmacy when they have identified an older adult at increased risk for falling.
- We request you review the pharmacist's recommendations and send your response back to the pharmacy.

How it works:

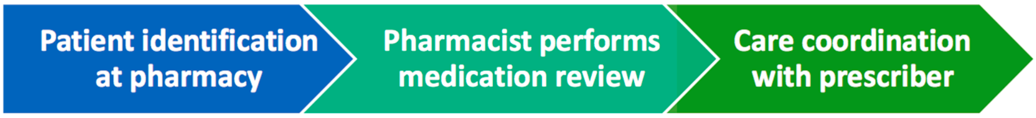

Figure S2: High-risk medication algorithms

Anticonvulsant Algorithm for Evaluating the Risk for Falls

The adverse effects associated with anticonvulsants may increase an individual's risk for falling. These agents cause sedation and dizziness resulting in the impairment of one's gait and balance and these effects are more pronounced in the elderly. Therefore, they should be used with caution in this population, especially when an individual is at increased risk for falls. In studies, anticonvulsants as a class have been found to increase the risk for falls and fracture. Even suggested alternatives may increase fall risk but are generally more tolerable and less likely to have altered pharmacokinetics in elderly patients compared to others in the class. Seizures may be controlled with lower or "subtherapeutic" doses of anticonvulsants in older patients. [1-7]

Brivaracetam (Briviact)
Carbamazepine (Tegretol, Carbatrol)
Clobazam (Onfi)
Divalproex sodium (Depakote)
Eslicarbazepine (Aptiom)
Ethosuximide (Zarotin)
Ezogabine (Potiga)
Felbamate (Felbatol)
Fosphenytoin (Cerebryx)
Gabapentin (Neurontin)
Lacosamide (Vimpat)
Lamotrigine (Lamictal)
Levetiracetam (Keppra)
Methsuximide (Celontin)
Oxcarbazepine (Trileptal)
Perampanel (Fycompa)
Phenobarbital
Phenytoin (Dilantin)
Pregabalin (Lyrica)
Primidone (Mysoine)
Rufinamide (Banzel)
Tiagabine (Gabtril)
Topiramate (Topamax)
Trimethadione (Tridione)
Valproate (Depakene)
Vigabatrin (Sabril)
Zonisamide (Zonegran)

Concerns

Several anticonvulsants can be problematic for the older population due to their potential for causing adverse effects. The adverse effects associated with the drugs may increase an individuals risk for falling.
Carbamazepine can cause sedation, neutropenia, and hyponatremia.
Divalproex sodium can cause tremor, sedation, parkinsonism, and hearing loss.
Phenobarbital can cause ataxia, memory problems, and sedation.
Phenytoin can cause ataxia, osteopenia, and sedation.

Consider addition of calcium and vitamin D if on anticonvulsant chronically.

Suggested Alternatives for Seizures

Alternatives depend on the type of seizure. Use the lowest possible strength for seizures protection. Monitor serum concentrations of medications.

Preferred initial agent for all seizure types in the elderly
Lamotrigine: start at 25mg/day, can increase by 25mg/day every 2 weeks; max 100-300mg/day

Other alternatives as adjunct or for select seizure types
Levetiracetam: start 500mg q12h, can increase by 500mg/day every 2 weeks; max 1500mg q12h. Requires renal dose reduction.
Gabapentin: start 300mg TID; max 600mg q8-12 hours. Requires renal dose reduction.

Suggested Alternatives for Neuropathic Pain/Chronic Pain

Must weigh benefit of treating pain with increased risk of falls.

Preferred agents
Includes duloxetine, venlafaxine, gabapentin, and pregabalin. May also consider topical agents including lidocaine patch and capsaicin if localized.

Refer to algorithm for tricyclic antidepressants

Suggested Approach for Changing Anticonvulsants or Discontinuing Therapy

Changing Anticonvulsants

The new anticonvulsant should be within therapeutic concentration before tapering the old one. It may take up to a year to taper an anticonvulsant during discontinuation or crossovers.

If seizures get worse, you may have to revert back to the previous dose and slow down the taper. If adverse effects occur, lowering the dose of the previous medication may help the patient tolerate the new anticonvulsant.

Patients should not drive during the taper and for awhile after.

Discontinuing Therapy

Consider slowly tapering patients off the seizure medication if they meet the below criteria.
(1) Seizure-free >2 years with subtherapeutic concentrations
(2) Taking the medication for a long time and were placed on anticonvulsants prophylactically or for a few seizures, especially after stroke, neurosurgery or head trauma.

UNC ESHELMAN SCHOOL OF PHARMACY

July 2017

Antidepressant Algorithm for Evaluating Risk for Falls

It is unclear how antidepressants increase an individuals risk for falling. Possible mechanisms include their potential to cause sedation and postural disturbances, although these effects vary with each agent and each person. Additionally, antidepressants may be indirectly associated with fall risk attributed to factors such as poor health status, depression, and weight loss. In studies, antidepressants have been found to increase the risk for falls and fracture.[8-10]

Selective Serotonin Reuptake Inhibitors
- Citalopram (Celexa)
- Escitalopram (Lexapro)
- Fluoxetine (Prozac)
- Mirtazapine (Remeron)
- Paroxetine (Paxil)
- Sertraline (Zoloft)

Serotonin Norepinephrine Reuptake Inhibitors
- Desvenlafaxine (Pristiq)
- Duloxetine (Cymbalta)
- Levomilnacipran (Fetzima)
- Venlafaxine (Effexor, Effexor XR)

Anxiolytics
- Buspirone (Buspar)

5-HT2 Receptor Antagonists
- Nefazodone (Serzone)
- Trazodone (Desyrel)

Noradrenergic Agonist
- Fluvoxamine (Luvox)
- Vilazodone (Viibryd)

Dopamine Reuptake Blocking Agents
- Bupropion (Wellbutrin, Wellbutrin SR)

Monoamine Oxidase Inhibitors
- Isocarboxazid (Marplan)
- Phenelzine (Nardil)
- Tranylcypromine (Parnate)**Anxiolytic**

Tricyclic Antidepressants *Refer to TCA Algorithm*

General Considerations

AVOID

Paroxetine due to greater anticholinergic properties than other antidepressants, which may increase one's risk for falling. Anticholinergic adverse effects include sedation, confusion, dizziness, gait and balance problems, and weakness.

AVOID

Fluoxetine due to long-half life, which may be even more pronounced in the elderly; thereby increasing the risk for excessive CNS stimulation, sleep disturbances, and increasing agitation.

AVOID

Fluvoxamine due to drug interactions and availability of effective and safer agents.

AVOID

Nefazodone, while not directly linked to falls, is associated with hepatotoxicity and significant drug interactions, which limit its use. Alternatives exist that are safer and as effective for treating depression.

AVOID

Isocarboxazid, **Phenelzine**, and **Tranylcypromine** should be avoided in the elderly due to their potential for toxicity and risk of drug-drug and drug-food interactions.

Must weigh benefit of treating depression with increased risk for falls associated with antidepressants. Selection of an antidepressant should be individualized, taking into account patient factors and concomitant medical conditions and medications.

Suggested Alternatives

Citalopram: start 10mg daily; max 20mg/day
Escitalopram: start 5mg daily; max 10mg/day
Sertraline: start 25mg daily; max 200mg/day
Duloxetine: avoid if GFR <30mL/min; start 30mg daily x2 weeks, increase to 60mg daily; max 120mg/day
Venlafaxine: start 37.5mg (XR) or 25mg once or twice daily (IR); max 225mg/day
Bupropion: start 37.5mg BID (IR), 100mg daily (SR), 150mg daily (XR); max 450mg/day (IR, XR), 400mg/day (SR)
Buspirone: as adjunct start 7.5mg daily; max 7.5mg BID

Educate patient on the potential for increased sedation, dizziness, and postural changes from the antidepressant.
Monitor closely for adverse effects and falls. Consider switching agent if adverse effects are apparent.
There is no one antidepressant or class considered the agent or class of choice in reducing one's risk for falls.
The association with antidepressants and fall risk has been attributed to all antidepressant agents.

UNC ESHELMAN SCHOOL OF PHARMACY

Antihypertensive Algorithms for Evaluating the Risk for Falls

There is mixed evidence regarding association of antihypertensives and fall risk. Hypotension and orthostatic hypotension may contribute to fall risk, but evidence is also inconsistent in this aspect. There is no strong evidence indicating a specific class is preferred over others due to lower fall risk. However, with the possibility of orthostatic hypotension contributing to falls and strong evidence of cardiovascular benefits with specific classes of antihypertensives, some may be preferred over others.[11-20]

Peripheral alpha-1 blockers	Calcium channel blockers	Diuretics	Beta-blockers
Doxazosin	Amlodipine	Amiloride	Acebutaolol
Prazosin	Diltiazem	Bumetanide	Atenolol
Terazosin	Felodipine	Chlorthalidone	Bisoprolol
	Isradipine	Chlorthiazide	Carvedilol
	Nicardipine	Eplerenone	Labetalol
Centrally-acting Medications	Nifedipine	Furosemide	Metoprolol
Clonidine	Nimodipine	Hydrochlorothiazide	Nadolol
Guanabenz	Nisoldipine	Indapamide	Nebivolol
Guanfacine	Verapamil	Metolazone	Penbutolol
Methyldopa		Spironolactone	Pindolol
Reserpine	Direct arterial vasodilators	Triamterene	Propranolol
	Hydralazine	Torsemide	Timolol
	Minoxidil		

General Considerations

AVOID

Peripheral alpha-1 blockers for treatment of hypertension due to high risk of orthostatic hypotension and availability of alternative agents with superior risk-benefit profile.

AVOID

Centrally-acting medications due to high risk of adverse CNS effects, bradycardia, and orthostatic hypotension.

AVOID

Immediate release nifedipine due to potential for hypotension

Suggested Alternatives

There is no clear evidence indicating that one medication or medication class should be preferred over others to reduce fall risk.

Selection of agents depends on patient's comorbid conditions. Generally, angiotensin-converting enzyme inhibitors, angiotensin II receptor blockers, calcium channel blockers, or thiazide diuretics would be preferred first-line agents for hypertension based on current guidelines.

Consider beta-blocker if patient has another compelling indication for its use or has resistant hypertension on preferred first-line agents. Selective beta-blockers (acebutolol, atenolol, betaxolol, bisoprolol, metoprolol, nebivolol) may have lower fall risk than non-selective beta blockers.

Antipsychotic Algorithm for Evaluating the Risk for Falls

Antipsychotics are thought to increase one's risk for falls due to their potential to cause significant adverse effects, including reduced alertness, impaired neuromuscular functioning, sedation, dizziness, postural hypotension, altered gait and balance, and extrapyramidal symptoms. In studies, antipsychotics have been found to increase one's risk for falls. Although atypical antipsychotics are generally better-tolerated overall and have less extrapyramidal effects, they are also associated with increased risk of falls. Avoid use of antipsychotics for treatment of conditions other than psychiatric conditions.[21-28]

Typical Antipsychotics		Atypical Antipsychotics	
Chlorpromazine (Thorazine)	Pimozide (Orap)	Aripiprazole (Abilify)	Olanzapine/Fluoxetine (Symbyax)
Fluphenazine (Permitil, Prolixin)	Thioridazine (Mellaril)	Asenapine Maleate (Sapris)	Paliperidone (Invega)
Haloperidol (Haldol)	Thiothixene (Navane)	Clozapine (Clozaril)	Quetiapine (Seroquel)
Loxapine (Loxitane, Loxitane C)	Trifluoroperazine (Stelazine)	Iloperidone (Fanapt)	Risperidone (Risperdal)
Molindone (Moban)		Lurasidone (Latuda)	Ziprasidone (Geodon)
Perphenazine (Trilafon)		Olanzapine (Zyprexa)	

General Considerations

AVOID

Thioridazine due to potential for increased CNS and extrapyramidal adverse effects. This drug has a high incidence of sedation, orthostatic hypotension, and anticholinergic adverse effects, which may increase one's risk for falls.

AVOID

Chlorpromazine due to a high incidence of sedation, orthostatic hypotension, and anticholinergic adverse effects, which may increase one's risk for falls.

AVOID

Antipsychotics in elderly individuals with dementia which has been associated with increased mortality. If required use lowest dose for shortest duration needed.

Insomnia

If low-dose antipsychotic being used, can discontinue without tapering.

Suggested Alternatives

The following agents should only be used when all possible reasons for insomnia have been ruled out and behavioral approaches to sleep management (i.e., sleep hygiene) have been addressed. The lowest dose possible for a short-term period is recommended.

Preferred drugs include: melatonin, ramelteon, trazodone, mirtazapine

Refer to algorithm for sedative hypnotics.

Behavioral Complications in Dementia

When discontinuing, consider tapering by 25% of original dose every 1-2 weeks.

Non-pharmacological interventions should be utilized before starting antipsychotic.

Risks and benefits of used should be carefully assessed.

If non-pharmacological approaches have failed and symptoms are severe, dangerous, and/or cause significant distress to patient, low dose, less anticholinergic agent may be acceptable for shortest duration possible. Consider trial discontinuation within 4 months.

Suggested Alternatives

Preferred drugs include:

Aripiprazole: start 2-5mg/day, can increase every 2 weeks if needed; max 30mg/day

Olanzapine: start 2.5mg/day; max 10mg/day

Quetiapine: start 12.5-25mg/day; max 200mg/day in 1-2 doses

Risperidone: start 0.25mg/day; max 6mg/day in 1-2 doses

Other Indications

Rule out any other causes of symptoms prior to initiating drug therapy.

For psychiatric conditions such as schizophrenia, schizoaffective disorder, bipolar disorder atypical antipsychotics with less anticholinergic properties may be preferred.

Suggested Alternatives

Preferred drugs include: aripiprazole, olanzapine, quetiapine, and risperidone

For management of acute psychiatric conditions such as delirium, address any contributing factors and utilize non-pharmacological interventions prior to medications. The previously noted medications may be used.

UNC ESHELMAN SCHOOL OF PHARMACY

Antispasmodic Algorithm for Evaluating the Risk for Falls

Antispasmodics have not been studied in association with increasing fall risk; however, the adverse effects associated with the drugs may increase an individual's risk for falling. These agents are highly anticholinergic and cause sedation, confusion, dizziness, gait and balance problems, and weakness. These effects are more pronounced in the elderly. Therefore, they should be used with caution in this population, especially when an individual is at an increased risk for falls.[29-35]

Skeletal Muscle Relaxants

Baclofen (Lioresal)
Carisoprodol (Soma)
Chlorzoxazone (Paraflex)
Cyclobenzaprine (Flexeril)
Dantrolene (Dantrium)
Metaxalone (Skelaxin)
Methocarbamol (Robaxin)
Orphenadrine (Norflex)
Tizanidine (Zanaflex)

Gastrointestinal Antispasmodics

Belladonna Alkaloids (Donnatol, others)
Clidinium-Chlordiazepoxide (Librax)
Dicyclomine (Bentyl)
Hyoscyamine (Levsin, Levsinex)
Propantheline (Pro-Banthine)

Urinary Antispasmodics

Darifenacin (Enablex)
Fesoterodine (Toviaz)
Flavoxate (Urispas)
Oxybutynin (Ditropan)
Solifenacin (Vesicare)
Tolterodine (Detrol)
Trospium (Sanctura)

General Considerations

AVOID MOST AGENTS

The benefit of using one of these agents in an elderly individual, especially an individual already at an increased risk for falling, will likely not outweigh the risks and adverse effects associated with these agents.

These agents are not recommended to be used in the elderly due to their potential for causing significant adverse effects. While these agents have not been studied in association with increasing fall risk, the adverse effects associated with the drugs may increase an individual's risk for falling. They are highly anticholinergic and cause sedation, confusion, dizziness, gait and balance problems, and weakness. Additionally, their effectiveness at doses tolerated by the elderly is questionable.

Spasms or Pain Associated with Muscle Spasms

Consider nonpharmacologic approaches, such as exercise and/or physical therapy, if appropriate.

If patient has true spasticity and the decision is made to use one of these agents in an elderly individual at an increased risk for falls, the following may be considered:

Suggested Alternatives

Baclofen: start 5mg 2-3x/day; max 80mg/day

Use the lowest dose possible for shortest duration.
Limit use to 2-3 weeks.
Document need for medication in light of fall risk.

Spasms Associated with Neurogenic Bladder or Urinary Incontience

Nonpharmacologic treatment should be first-line prior to trying medication. All agents have similar efficacy.

Some newer agents and topical agents have less CNS effects and may be preferred over other agents. The following may be considered:

Suggested Alternatives

Darifenacin: 7.5mg daily, can increase to 15mg after at least 2 weeks
Fesoterodine: 4mg daily; max 8mg/day
Trospium: 20mg BID (IR) or 60mg daily (XR)
Oxybutynin Transdermal Patch: apply one 3.9mg patch q3-4 days

UNC ESHELMAN SCHOOL OF PHARMACY

July 2017

Benzodiazepine Algorithm for Evaluating the Risk for Falls

The adverse effects associated with benzodiazepines may increase an individual's risk for falling. These agents are highly anticholinergic and cause sedation, confusion, dizziness, gait and balance problems, and weakness. These effects are more pronounced in the elderly. Therefore, they should be used with caution in this population, especially when an individual is at increased risk for falls. In studies, benzodiazepines as a class have been found to increase the risk for falls and fracture.[36-39]

- Alprazolam (Xanax)
- Chlorazepate (Tranxene)
- Chlordiazepoxide (Librium, Limbitrol, Librax)
- Clonazepam (Klonopin)
- Diazepam (Valium)
- Estazolam (Eurodin)
- Eszopiclone (Lunesta)
- Flurazepam (Dalmane)
- Lorazepam (Ativan)
- Oxazepam (Serax)
- Quazepam (Doral)
- Temazepam (Restoril)
- Triazolam (Halcion)
- Zaleplon (Sonata)
- Zolpidem (Ambien)

Anxiety

All suggested alternatives may increase a patient's fall risk. One must determine the risk versus the benefit when selecting an alternative.

Suggested Alternatives

SSRIs [Sertraline (Zoloft), Escitalopram (Lexapro), Citalopram (Celexa)], SNRIs [Venlafaxine (Effexor), and Duloxetine (Cymbalta)], and Buspirone (Buspar), can be used to treat anxiety and they are not associated with the same degree of CNS depression as benzodiazepines.

Refer to algorithm for antidepressants.

In addition, cognitive-behavioral therapy has been shown to be effective in the management of generalized anxiety disorder.

Insomnia

The following agents should only be used when all possible reasons for insomnia have been ruled out and behavioral approaches to sleep management (i.e., sleep hygiene) have been addressed.

Suggested Alternatives

Melatonin: start at 1mg QHS; max 10mg/night or Ramelteon (Rozerem): start at 8mg QHS; max 8mg/night,

Trazodone (Desyrel): start 25mg QHS; max 100mg/night, or Mirtazapine (Remeron): 7.5mg QHS; max 15mg/night if has concomitant depression

Refer to algorithm for sedative-hypnotics.

Other Indications

Consider re-evaluating need/indication for the benzodiazepine due to potential for adverse events, especially falls. It is likely that the risk associated with these agents outweighs any benefit.

Suggested Alternatives

If benzodiazepine is required, lorazepam, oxazepam, temazepam may be preferred because metabolism is not affected by impaired liver function and they do not have active metabolites

The lowest dose possible for a short-term period is recommended.

Tapering Considerations

Slow tapering recommended when stopping benzodiazepines.

Consider decreasing dose by 25% every two weeks, and if possible, 12.5% reductions near end of taper and/or planned drug-free days. If dosage form doesn't allow for 25% reduction, consider using 50% reduction initially and then drug-free days in the latter part of tapering.

If symptoms relapse, can consider maintaining current dose for 1-2 weeks then resume taper at slow rate.

UNC
ESHELMAN
SCHOOL OF PHARMACY

July 2017

Opioid Algorithm for Evaluating the Risk for Falls

The opioids likely increase an individual's risk for falling due to their potential for causing adverse effects, including reduced alertness, impaired neuromuscular function, sedation, dizziness, impaired cognition, and unsteadiness or impaired functioning. In studies, opioids/narcotics have been found to increase one's risk for falls and fracture, although findings are inconsistent.[45-49]

- Buprenorphine (Butrans)
- Codeine (Tylenol with Codeine)
- Fentanyl (Duragesic)
- Hydrocodone (Norco, Vicodin, Lorcet)
- Hydromorphone (Dilaudid)
- Levorphanol
- Meperidine (Demerol)
- Methadone (Methadose)
- Morphine (MS Contin)
- Oxycodone (Oxycontin, Roxicodone)
- Oxymorphone (Opana; Opana ER)
- Pentazocine/naloxone
- Tapentadol (Nucynta, Nucynta ER)

Note: The above agents are found in various combination products

General Considerations

AVOID

Pentazocine/naloxone as it causes CNS adverse effects, including confusion and hallucinations, which may increase one's risk for falls.

AVOID

Meperidine as it is not an effective oral analgesic in dosages commonly used and may have a higher risk of neurotoxicity, which may increase one's risk for falls.

Nociceptive Pain

Must weigh benefit of treating pain and increased risk of adverse effects and falls associated with opioids. If the opioid is continued, educate patient on the potential for increased sedation, dizziness, unsteadiness, and confusion, and closely monitor for the presence of these adverse effects.

Consider the following:
Limit dose to 1 tablet at a time rather than 1-2 tablets.
Switch drug if adverse effects are apparent.

Suggested Alternatives

Localized Pain:
Topical Capsaicin: usually applied 2-4 times daily
Diclofenac gel (Voltaren): 2-4g up to 4 times daily; max 32g/day

Mild-Moderate pain:
Acetaminophen (Tylenol): dose q6-8hrs; max 3g/day
Salsalate (Salflex): 500mg q8-12h; max 3g/day
NSAIDs (ibuprofen, naproxen, diclofenac, celecoxib) use with caution if no HF and eGFR >30mL/min and given with PPI for gastroprotection. Note: avoid indomethacin due to CNS adverse effects; avoid ketorolac due to increased risk of bleeding, renal failure, high blood pressure, and heart failure.

Moderate-Severe pain:
Tramadol (Ultram): avoid if CrCl <30mL/min, start 25mg (IR) QHS; max 300mg/day (divided QID)
Oxycodone: 2.5mg QHS; max 2.5-5mg q4-6h
Morphine sulfate: 7.5mg QHS; max15mg q12h
Increase slowly and use the lowest dose possible to control pain.

Neuropathic Pain

All suggested oral alternatives may increase a patient's fall risk. One must determine the risk versus the benefit when selecting an alternative.

Suggested Alternatives

Topical Agents:
Capsaicin: usually applied 2-4 times daily
Lidocaine patch (Lidoderm): apply to affected area for 12 hours, then remove for 12 hours

Oral Agents:
Duloxetine (Cymbalta): avoid if GFR <30mL/min, start 30mg daily; max 60mg/day

Venlafaxine (Effexor): start 37.5mg daily; max 225mg/day

Gabapentin (Neurontin): must be renally adjusted, start 100mg QHS, then 100mg q8h; max 3600mg/day
Pregabalin (Lyrica): must be renally adjusted, start 50mg QHS, then 50mg q8h; max 300mg/day

Refer to algorithm for TCAs

UNC ESHELMAN SCHOOL OF PHARMACY

July 2017

Sedative Hypnotic Algorithm for Evaluating the Risk for Falls

The adverse effects associated with sedative hypnotics may increase an individual's risk for falling. These agents are highly anticholinergic and cause sedation, confusion, dizziness, gait and balance problems, and weakness. These effects are more pronounced in the elderly. Therefore, they should be used with caution in this population, especially when an individual is at increased risk for falls. In studies, sedative hypnotics as a class have been found to increase the risk for falls and fracture.[50-54]

Amobarbital (Amytal)
Butabarbital
Butalbital
Diphenhydramine
Doxylamine
Meprobamate (Miltown, Equanil)
Pentobarbital (Nembutal)
Secobarbital (Seconal)

Anxiety

Suggested Alternatives

All suggested alternatives may increase a patient's fall risk. One must determine the risk versus the benefit when selecting an alternative.

SSRI/SNRIs can be used to treat anxiety. Preferred agents include duloxetine, venlafaxine, sertraline, escitalopram, and buspirone.

Refer to algorithm for antidepressants.

May consider short term use of benzodiazepines (lorazepam, oxazepam, temazepam) for severe anxiety that has not responded to preferred agents.

Refer to algorithm for benzodiazepines.

Insomnia

Suggested Alternatives

The following agents should only be used when all possible reasons for insomnia have been ruled out and behavioral approaches to sleep management (i.e., sleep hygiene) have been addressed.

Melatonin: start at 1mg QHS; max 10mg/night or Ramelteon (Rozerem): start at 8mg QHS; max 8mg/night, Trazodone (Desyrel): start 25mg QHS; max 100mg/night, or Mirtazapine (Remeron): 7.5mg QHS; max 15mg/night if has concomitant depression

AVOID

Diphenhydramine-containing products (e.g., Tylenol PM, Benadryl, Nytol, Sominex) and doxylamine-containing products (e.g., Unisom Nightime). These agents are highly anticholinergic and cause sedation, confusion, dizziness, gait and balance problems, and weakness. Additionally, their effectiveness for a sleep is questionable.

Seizure

Suggested Alternatives

Newer anticonvulsants lamotrigine, levetiracetam, and gabapentin are preferred in elderly patients due to improved safety and better tolerability.

Refer to algorithm for anticonvulsants.

UNC
ESHELMAN
SCHOOL OF PHARMACY

Tricyclic Antidepressant Algorithm for Evaluating the Risk for Falls

The tricyclic antidepressants are associated with high incidence of anticholinergic adverse effects, including reduced alertness, impaired neuromuscular functioning, sedation, dizziness, postural hypotension, altered gait and balance, and confusion. In studies, the tricyclic antidepressants have been associated with increased risk of falls.[55-59]

Amitriptyline (Elavil)
Amoxapine (Asendin)
Clomipramine (Anafranil)
Desipramine (Norpramine)
Doxepine (Sinequan)
Imipramine (Tofranil)
Maprotiline (Ludiomil)
Nortriptyline (Pamelor)
Protriptyline (Vivactil)
Trimipramine (Surmontil)

Depression

Suggested Alternatives
Citalopram (Celexa)
Sertraline (Zoloft)
Escitalopram (Lexapro)
Bubpropion (Wellbutrin)
Venlafaxine (Effexor)
Duloxetine (Cymbalta)

AVOID
Paroxetine (Paxil)
Fluoxetine (Prozac)
Nefazodone (Serzone)
Fluvoxamine (Luvox)

AVOID
TCAs due to potential for strong anticholinergic and sedating properties which increase fall risk.

AVOID
Isocarboxazide Marplan)
Phenelzine (Nardil)
Tranylcypromine (Parnate)

Refer to algorithm for antidepressants

Neuropathic Pain/ Chronic Pain

Must weigh benefit of treating pain with increased risk for falls.

Suggested Alternatives
Duloxetine (Cymbalta): avoid if GFR <30 mL/min, start 30mg daily; max 60mg/day
Venlafaxine (Effexor): start 37.5mg daily; max 225mg/day
Gabapentin: must be renally adjusted; start 50mg QHs, then 50mg q8h; max 300mg/day
Lidocaine patch: apply to affected area for 12 hrs, then remove for 12 hrs
Other topical lidocaine: usually applied 3-4 times daily
Capsaicin topical: usually applied 2-4 times daily

If a TCA is used and effectiveness has been demonstrated, ensure that the individual is on the lowest dose possible to control the pain and minimize adverse events.

If a TCA is needed, consider nortriptyline (max 30-50mg/day) or desipramine (max 150mg/day).

Refer to algorithm for opioids

Insomnia

Suggested Alternatives
The following agents should only be used when all possible reasons for insomnia have been ruled out and behavioral approaches to sleep management (i.e., sleep hygiene) have been addressed. The lowest dose possible for a short-term period is recommended.

Preferred drugs are: melatonin, ramelteon, trazodone, mirtazapine

AVOID
Avoid diphenhydramine containing products (e.g., Tylenol PM, Benadryl) and doxylamine-containing products (e.g., Unisom Sleeptabs). These agents are highly anticholinergic and cause sedation, confusion, dizziness, gait and balance problems, and weakness. Additionally, their effectiveness for sleep is questionable.

Refer to algorithm for sedative-hypnotics

Other Indications

Suggested Alternatives
Consider re-evaluating need/indication for the TCA due to potential for adverse events, especially falls. It is likely that the risk associated with this agent outweighs any benefit.

UNC ESHELMAN SCHOOL OF PHARMACY

July 2017

High-risk medication algorithm references

1. American Geriatrics Society 2015 Beers Criteria Update Expert Panel. American Geriatrics Society 2015 Updated Beers Criteria for Potentially Inappropriate Medication Use in Older Adults. Journal of the American Geriatrics Society. 2015;63(11):2227-2246.
2. Woolcott JC, Richardson KJ, Wiens MO, et al. Meta-analysis of the impact of 9 medication classes on falls in elderly persons. Arch Intern Med. 2009;169(21):1952-1960.

3. Maximos M, Chang F, Patel T. Risk of falls associated with antiepileptic drug use in ambulatory elderly populations: A systematic review. Canadian pharmacists journal : CPJ = Revue des pharmaciens du Canada : RPC. 2017;150(2):101-111.
4. Boyle N, Naganathan V, Cumming RG. Medication and falls: risk and optimization. Clinics in geriatric medicine. 2010;26(4):583-605.
5. Hanlon JT, Semla TP, Schmader KE. Alternative Medications for Medications in the Use of High-Risk Medications in the Elderly and Potentially Harmful Drug–Disease Interactions in the Elderly Quality Measures. Journal of the American Geriatrics Society. 2015;63(12):e8-e18.
6. Schmidt D, Schachter SC. Drug treatment of epilepsy in adults. Bmj. 2014;348:g254.
7. Pohlmann-Eden B, Marson AG, Noack-Rink M, et al. Comparative effectiveness of levetiracetam, valproate and carbamazepine among elderly patients with newly diagnosed epilepsy: subgroup analysis of the randomized, unblinded KOMET study. BMC neurology. 2016;16(1):149.
8. American Geriatrics Society 2015 Beers Criteria Update Expert Panel. American Geriatrics Society 2015 Updated Beers Criteria for Potentially Inappropriate Medication Use in Older Adults. Journal of the American Geriatrics Society. 2015;63(11):2227-2246.
9. Hanlon JT, Semla TP, Schmader KE. Alternative Medications for Medications in the Use of High-Risk Medications in the Elderly and Potentially Harmful Drug–Disease Interactions in the Elderly Quality Measures. Journal of the American Geriatrics Society. 2015;63(12):e8-e18.
10. The Management of MDD Working Group. VA/DoD Clinical Practice Guideline for the Management of Major Depressive Disorder. Washington, DC: U.S. Department of Veterans Affairs and Department of Defense, 2016.
11. American Geriatrics Society 2015 Beers Criteria Update Expert Panel. American Geriatrics Society 2015 Updated Beers Criteria for Potentially Inappropriate Medication Use in Older Adults. Journal of the American Geriatrics Society. 2015;63(11):2227-2246.
12. Woolcott JC, Richardson KJ, Wiens MO, et al. Meta-analysis of the impact of 9 medication classes on falls in elderly persons. Arch Intern Med. 2009;169(21):1952-1960.
13. Zang G. Antihypertensive drugs and the risk of fall injuries: a systematic review and meta-analysis. The Journal of international medical research. 2013;41(5):1408-1417.
14. Lipsitz LA, Habtemariam D, Gagnon M, et al. Reexamining the Effect of Antihypertensive Medications on Falls in Old Age. Hypertension (Dallas, Tex : 1979). 2015;66(1):183-189.
15. Hartog LC, Cimzar-Sweelssen M, Knipscheer A, et al. Orthostatic hypotension does not predict recurrent falling in a nursing home population. Archives of gerontology and geriatrics. 2017;68:39-43.
16. Marcum ZA, Perera S, Newman AB, et al. Antihypertensive Use and Recurrent Falls in Community-Dwelling Older Adults: Findings From the Health ABC Study. The journals of gerontology Series A, Biological sciences and medical sciences. 2015;70(12):1562-1568.
17. Margolis KL, Palermo L, Vittinghoff E, et al. Intensive blood pressure control, falls, and fractures in patients with type 2 diabetes: the ACCORD trial. Journal of general internal medicine. 2014;29(12):1599-1606.
18. Tinetti ME, Han L, Lee DS, et al. Antihypertensive medications and serious fall injuries in a nationally representative sample of older adults. JAMA internal medicine. 2014;174(4):588-595.
19. Ham AC, van Dijk SC, Swart KMA, et al. Beta-blocker use and fall risk in older individuals: Original results from two studies with meta-analysis. British journal of clinical pharmacology. 2017.
20. Parekh N, Page A, Ali K, Davies K, Rajkumar C. A practical approach to the pharmacological management of hypertension in older people. Therapeutic advances in drug safety. 2017;8(4):117-132.
21. American Geriatrics Society 2015 Beers Criteria Update Expert Panel. American Geriatrics Society 2015 Updated Beers Criteria for Potentially Inappropriate Medication Use in Older Adults. *Journal of the American Geriatrics Society.* 2015;63(11):2227-2246.
22. Hien le TT, Cumming RG, Cameron ID, et al. Atypical antipsychotic medications and risk of falls in residents of aged care facilities. *Journal of the American Geriatrics Society.* 2005;53(8):1290-1295.
23. Huang AR, Mallet L, Rochefort CM, Eguale T, Buckeridge DL, Tamblyn R. Medication-related falls in the elderly: causative factors and preventive strategies. *Drugs Aging.* 2012;29(5):359-376.
24. Boyle N, Naganathan V, Cumming RG. Medication and falls: risk and optimization. *Clinics in geriatric medicine.* 2010;26(4):583-605.
25. Bjerre LM, Farrell B, Hogel M, et al. Deprescribing antipsychotics for behavioural and psychological symptoms of dementia (BPSD) and insomnia: an evidence-based clinical practice guideline. 2016.
26. Suzuki T, Remington G, Uchida H, Rajji TK, Graff-Guerrero A, Mamo DC. Management of schizophrenia in late life with antipsychotic medications: a qualitative review. *Drugs Aging.* 2011;28(12):961-980.
27. Wang LY, Borisovskaya A, Maxwell AL, Pascualy M. Common psychiatric problems in cognitively impaired older patients: causes and management. *Clinics in geriatric medicine.* 2014;30(3):443-467.
28. Reus VI, Fochtmann LJ, Eyler AE, et al. The American Psychiatric Association Practice Guideline on the Use of Antipsychotics to Treat Agitation or Psychosis in Patients With Dementia. *American Journal of Psychiatry.* 2016;173(5):543-546.
29. American Geriatrics Society 2015 Beers Criteria Update Expert Panel. American Geriatrics Society 2015 Updated Beers Criteria for Potentially Inappropriate Medication Use in Older Adults. *Journal of the American Geriatrics Society.* 2015;63(11):2227-2246.

30. Derner MM, Linhart CA, Pederson LM, et al. Injuries in Adults 65 Years of Age and Older Prescribed Muscle Relaxants. *The Consultant pharmacist : the journal of the American Society of Consultant Pharmacists.* 2016;31(9):511-517.
31. Spence MM, Karim FA, Lee EA, Hui RL, Gibbs NE. Risk of Injury in Older Adults Using Gastrointestinal Antispasmodic and Anticholinergic Medications. *Journal of the American Geriatrics Society.* 2015;63(6):1197-1202.
32. Spence MM, Shin PJ, Lee EA, Gibbs NE. Risk of injury associated with skeletal muscle relaxant use in older adults. *The Annals of pharmacotherapy.* 2013;47(7-8):993-998.
33. Marcum ZA, Duncan NA, Makris UE. Pharmacotherapies in Geriatric Chronic Pain Management. *Clinics in geriatric medicine.* 2016;32(4):705-724.
34. Chancellor M, Boone T. Anticholinergics for overactive bladder therapy: central nervous system effects. *CNS neuroscience & therapeutics.* 2012;18(2):167-174.
35. Chancellor MB, Staskin DR, Kay GG, Sandage BW, Oefelein MG, Tsao JW. Blood-brain barrier permeation and efflux exclusion of anticholinergics used in the treatment of overactive bladder. *Drugs Aging.* 2012;29(4):259-273.
36. Schroeck JL, Ford J, Conway EL, et al. Review of Safety and Efficacy of Sleep Medicines in Older Adults. *Clinical therapeutics.* 2016;38(11):2340-2372.
37. American Geriatrics Society 2015 Beers Criteria Update Expert Panel. American Geriatrics Society 2015 Updated Beers Criteria for Potentially Inappropriate Medication Use in Older Adults. *Journal of the American Geriatrics Society.* 2015;63(11):2227-2246.
38. Hanlon JT, Semla TP, Schmader KE. Alternative Medications for Medications in the Use of High-Risk Medications in the Elderly and Potentially Harmful Drug–Disease Interactions in the Elderly Quality Measures. *Journal of the American Geriatrics Society.* 2015;63(12):e8-e18.
39. Pottie K, Thompson W, Davies S, et al. Evidence-based clinical practice guideline for deprescribing benzodiazepine receptor agonists. Unpublished manuscript. 2016.
40. Nelson JM, Dufraux K, Cook PF. The relationship between glycemic control and falls in older adults. *Journal of the American Geriatrics Society.* 2007;55(12):2041-2044.
41. Johnston SS, Conner C, Aagren M, Ruiz K, Bouchard J. Association between hypoglycaemic events and fall-related fractures in Medicare-covered patients with type 2 diabetes. *Diabetes, obesity & metabolism.* 2012;14(7):634-643.
42. Signorovitch JE, Macaulay D, Diener M, et al. Hypoglycaemia and accident risk in people with type 2 diabetes mellitus treated with non-insulin antidiabetes drugs. *Diabetes, obesity & metabolism.* 2013;15(4):335-341.
43. American Geriatrics Society 2015 Beers Criteria Update Expert Panel. American Geriatrics Society 2015 Updated Beers Criteria for Potentially Inappropriate Medication Use in Older Adults. *Journal of the American Geriatrics Society.* 2015;63(11):2227-2246.
44. Hanlon JT, Semla TP, Schmader KE. Alternative Medications for Medications in the Use of High-Risk Medications in the Elderly and Potentially Harmful Drug–Disease Interactions in the Elderly Quality Measures. *Journal of the American Geriatrics Society.* 2015;63(12):e8-e18.
45. American Geriatrics Society 2015 Beers Criteria Update Expert Panel. American Geriatrics Society 2015 Updated Beers Criteria for Potentially Inappropriate Medication Use in Older Adults. *Journal of the American Geriatrics Society.* 2015;63(11):2227-2246.
46. Hanlon JT, Semla TP, Schmader KE. Alternative Medications for Medications in the Use of High-Risk Medications in the Elderly and Potentially Harmful Drug–Disease Interactions in the Elderly Quality Measures. *Journal of the American Geriatrics Society.* 2015;63(12):e8-e18.
47. Abdulla A, Adams N, Bone M, et al. Guidance on the management of pain in older people. *Age and ageing.* 2013;42(suppl_1):i1-i57.
48. Makris UE, Abrams RC, Gurland B, Reid MC. Management of persistent pain in the older patient: a clinical review. *JAMA : the journal of the American Medical Association.* 2014;312(8):825-836.
49. Fine PG. Treatment Guidelines for the Pharmacological Management of Pain in Older Persons. *Pain Medicine.* 2012;13(suppl_2):S57-S66.
50. American Geriatrics Society 2015 Beers Criteria Update Expert Panel. American Geriatrics Society 2015 Updated Beers Criteria for Potentially Inappropriate Medication Use in Older Adults. *Journal of the American Geriatrics Society.* 2015;63(11):2227-2246.
51. Hanlon JT, Semla TP, Schmader KE. Alternative Medications for Medications in the Use of High-Risk Medications in the Elderly and Potentially Harmful Drug–Disease Interactions in the Elderly Quality Measures. *Journal of the American Geriatrics Society.* 2015;63(12):e8-e18.
52. Abdulla A, Adams N, Bone M, et al. Guidance on the management of pain in older people. *Age and ageing.* 2013;42(suppl_1):i1-i57.
53. Makris UE, Abrams RC, Gurland B, Reid MC. Management of persistent pain in the older patient: a clinical review. *JAMA : the journal of the American Medical Association.* 2014;312(8):825-836.
54. Fine PG. Treatment Guidelines for the Pharmacological Management of Pain in Older Persons. *Pain Medicine.* 2012;13(suppl_2):S57-S66.
55. American Geriatrics Society 2015 Beers Criteria Update Expert Panel. American Geriatrics Society 2015 Updated Beers Criteria for Potentially Inappropriate Medication Use in Older Adults. Journal of the American Geriatrics Society. 2015;63(11):2227-2246.

56. Boyle N, Naganathan V, Cumming RG. Medication and falls: risk and optimization. Clinics in geriatric medicine. 2010;26(4):583-605.

57. Hanlon JT, Semla TP, Schmader KE. Alternative Medications for Medications in the Use of High-Risk Medications in the Elderly and Potentially Harmful Drug–Disease Interactions in the Elderly Quality Measures. Journal of the American Geriatrics Society. 2015;63(12):e8-e18.

58. Schroeck JL, Ford J, Conway EL, et al. Review of Safety and Efficacy of Sleep Medicines in Older Adults. Clinical therapeutics. 2016;38(11):2340-2372.

59. Makris UE, Abrams RC, Gurland B, Reid MC. Management of persistent pain in the older patient: a clinical review. JAMA : the journal of the American Medical Association. 2014;312(8):825-836.

Table S2. High-risk medication index.

Medication	Algorithm
**A**	
Abilify	Antipsychotics
acebutalol	Antihypertensives
Actiq	Opioids
Aldactazide	Antihypertensives
Aldactone	Antihypertensives
Aldomet	Antihypertensives
alprazolam	Benzodiazepines
Ambien	Benzodiazepines
amiloride	Antihypertensives
amitriptyline	Tricyclic Antidepressants
amlodipine	Antihypertensives
amobarbital	Sedative Hypnotics
amoxapine	Tricyclic Antidepressants
Amytal	Sedative Hypnotics
Anafranil	Tricyclic Antidepressants
Apresoline	Antihypertensives
Aptiom	Anticonvulsants
aripiprazole	Antipsychotics
asenapine maleate	Antipsychotics
Asendin	Tricyclic Antidepressants
Atacand HCT	Antihypertensives
atenolol	Antihypertensives
Ativan	Benzodiazepines
Avalide	Antihypertensives
Azor	Antihypertensives
**B**	
baclofen	Antispasmodics
Banzel	Anticonvulsants
belladonna alkaloids	Antispasmodics
Benicar HCT	Antihypertensives
Bentyl	Antispasmodics
bisoprolol	Antihypertensives

Blocadren	Antihypertensives
brivaracetam	Anticonvulsants
Briviact	Anticonvulsants
bumetanide	Antihypertensives
Bumex	Antihypertensives
buprenorphine	Opioids
bupropion	Antidepressants

Medication	Algorithm
Buspar	Antidepressants
buspirone	Antidepressants
butabarbital	Sedative Hypnotics
butalbital	Sedative Hypnotics
Butisol	Sedative Hypnotics
Butrans	Opioids
Bystolic	Antihypertensives
**C**	
Caduet	Antihypertensives
Calan	Antihypertensives
carbamazepine	Anticonvulsants
Carbatrol	Anticonvulsants
Cardene	Antihypertensives
Cardizem	Antihypertensives
Cardura	Antihypertensives
carisoprodol	Antispasmodics
Cartia	Antihypertensives
carvedilol	Antihypertensives
Catapres	Antihypertensives
Celexa	Antidepressants
Celontin	Anticonvulsants
Cerebryx	Anticonvulsants
chlorazepate	Benzodiazepines
chlordiazepoxide	Benzodiazepines
chlorthiazide	Antihypertensives
chlorpromazine	Antipsychotics
chlorthalidone	Antihypertensives
chlorzoxazone	Antispasmodics
citalopram	Antidepressants
clidinium-chloridazepoxide	Antispasmodics
clobazam	Anticonvulsants
clomipramine	Tricyclic Antidepressants
clonazepam	Benzodiazepines
clonidine	Antihypertensives
clozapine	Antipsychotics
Clozaril	Antipsychotics
codeine	Opioids
Coreg	Antihypertensives
Corgard	Antihypertensives
cyclobenzaprine	Antispasmodics
Cymbalta	Antidepressants

Medication	Algorithm
**D**	
Dalmane	Benzodiazepines
Dantrium	Antispasmodics
dantrolene	Antispasmodics
darifenacin	Antispasmodics
Demadex	Antihypertensives
Demerol	Opioids
Depakene	Anticonvulsants
Depakote	Anticonvulsants
desipramine	Tricyclic Antidepressants
desvenlafaxine	Antidepressants
Desyrel	Antidepressants
Detrol	Antispasmodics
diazepam	Benzodiazepines
dicyclomine	Antispasmodics
Dilacor	Antihypertensives
Dilantin	Anticonvulsants
Dilaudid	Opioids
diltiazem	Antihypertensives
Diltzac	Antihypertensives
Diovan HCT	Antihypertensives
diphenhydramine	Sedative Hypnotics
Ditropan	Antispasmodics
Diuril	Antihypertensives
divalproex sodium	Anticonvulsants
Dolophine	Opioids
Donnatol	Antispasmodics
Doral	Benzodiazepines
doxazosin	Antihypertensives
doxepin	Tricyclic Antidepressants
doxylamine	Sedative Hypnotics
duloxetine	Antidepressants
Duragesic	Opioids
Dyazide	Antihypertensives
Dynacirc	Antihypertensives
Dyrenium	Antihypertensives
**E**	
Effexor (Effexor XR)	Antidepressants
Elavil	Tricyclic Antidepressants
Enablex	Antispasmodics

Medication	Algorithm
eplerenone	Antihypertensives
Equanil	Sedative Hypnotics
escitalopram	Antidepressants
Esidrix	Antihypertensives
eslicarbazepine	Anticonvulsants
estazolam	Benzodiazepines
eszopiclone	Benzodiazepines
ethosuximide	Anticonvulsants
Exforge	Antihypertensives
ezogabine	Anticonvulsants
**F**	
Fanapt	Antipsychotics
felbamate	Anticonvulsants
Felbatol	Anticonvulsants
felodipine	Antihypertensives
fentanyl	Opioids
Fentora	Opioids
fesoterodine	Antispasmodics
Fetzima	Antidepressants
Fioricet	Sedative Hypnotics
Fiorinal	sedative hypotics
flavoxate	Antispasmodics
Flexeril	Antispasmodics
fluoxetine	Antidepressants & Antipsychotics
fluphenazine	Antipsychotics
flurazepam	Benzodiazepines
fluvoxamine	Antidepressants
fosphenytoin	Anticonvulsants
furosemide	Antihypertensives
Fycompa	Anticonvulsants
**G**	
gabapentin	Anticonvulsants
Gabitril	Anticonvulsants
Geodon	Antipsychotics
guanabenz	Antihypertensives
guanfacine	Antihypertensives
**H**	
Halcion	Benzodiazepines
Haldol	Antipsychotics
haloperidol	Antipsychotics

Medication	Algorithm
hydralazine	Antihypertensives
hydrochlorothiazide	Antihypertensives
hydrocodone	Opioids
Hydrodiuril	Antihypertensives
hydromorphone	Opioids
Hygroton	Antihypertensives
hyoscyamine	Antispasmodics
Hypovase	Antihypertensives
Hytrin	Antihypertensives
Hyzaar	Antihypertensives
**I**	
iloperidone	Antipsychotics
imipramine	Tricyclic Antidepressants
indapamide	Antihypertensives
Inderal	Antihypertensives
Innopran	Antihypertensives
Inspra	Antihypertensives
Intuniv	Antihypertensives
Invega	Antipsychotics
isocarboxazid	Antidepressants
Isoptin	Antihypertensives
isradipine	Antihypertensives
**K**	
Keppra	Anticonvulsants
Klonopin	Benzodiazepines
**L**	
labetalol	Antihypertensives
lacosamide	Anticonvulsants
Lamictal	Anticonvulsants
lamotrigine	Anticonvulsants
Lasix	Antihypertensives
Latuda	Antipsychotics
Lentopres	Antihypertensives
Levatol	Antihypertensives
Levbid	Antispasmodics
levetiracetam	Anticonvulsants
Levo-Dromoran	Opioids
levomilnacipran	Antidepressants
levorphanol	Opioids
Levsin	Antispasmodics

Medication	Algorithm
Levsinex	Antispasmodics
Lexapro	Antidepressants
Librax	Antispasmodics
Librax	Benzodiazepines
Librium	Benzodiazepines
Limbitrol	Benzodiazepines
Lioresal	Antispasmodics
Loniten	Antihypertensives
Lopressor	Antihypertensives
lorazepam	Benzodiazepines
Lorcet	Opioids
Lortab	Opioids
Lotrel	Antihypertensives
loxapine	Antipsychotics
Loxitane	Antipsychotics
Lozol	Antihypertensives
Ludiomil	Tricyclic Antidepressants
Lunesta	Benzodiazepines
lurasidone	Antipsychotics
Luvox	Antidepressants
Lyrica	Anticonvulsants
**M**	
maprotiline	Tricyclic Antidepressants
Marplan	Antidepressants
Maxzide	Antihypertensives
Mellaril	Antipsychotics
meperidine	Opioids
meprobamate	Sedative Hypnotics
metaxalone	Antispasmodics
methadone	Opioids
methocarbamol	Antispasmodics
methsuximide	Anticonvulsants
methyldopa	Antihypertensives
metolazone	Antihypertensives
metoprolol	Antihypertensives
Microzide	Antihypertensives
Midamor	Antihypertensives
Miltown	Sedative Hypnotics
Minipress	Antihypertensives
minoxidil	Antihypertensives
mirtazapine	Antidepressants

Medication	Algorithm
Moban	Antipsychotics
molindone	Antipsychotics
morphine	Opioids
MS Contin	Opioids
Mysoline	Anticonvulsants
**N**	
nadalol	Antihypertensives
Nardil	Antidepressants
Navane	Antipsychotics
Nebilet	Antihypertensives
nebivolol	Antihypertensives
nefazodone	Antidepressants
Nembutal	Sedative Hypnotics
Neurontin	Anticonvulsants
nicardipine	Antihypertensives
Nifedipine	Antihypertensives
nimodipine	Antihypertensives
Nimotop	Antihypertensives
nisoldipine	Antihypertensives
Norco	Opioids
Norflex	Antispasmodics
Normodyne	Antihypertensives
Norpramine	Tricyclic Antidepressants
nortriptyline	Tricyclic Antidepressants
Norvasc	Antihypertensives
Nucynta	Opioids
**O**	
olanzapine	Antipsychotics
Onfi	Anticonvulsants
Opana	Opioids
Orap	Antipsychotics
Oretic	Antihypertensives
orphenadrine	Antispasmodics
oxazepam	Benzodiazepines
oxcarbazepine	Anticonvulsants
oxybutynin	Antispasmodics
oxycodone	Opioids
OxyContin	Opioids
oxymorphone	Opioids
**P**	

Medication	Algorithm
paliperidone	Antipsychotics
Pamelor	Tricyclic Antidepressants
Paraflex	Antispasmodics
Parnate	Antidepressants
paroxetine	Antidepressants
Paxil	Antidepressants
penbutolol	Antihypertensives
pentobarbital	Sedative Hypnotics
pentazocine	Opioids
perampanel	Anticonvulsants
Percocet	Opioids
Permitil	Antipsychotics
perphenazine	Antipsychotics
phenelzine	Antidepressants
phenobarbital	Anticonvulsants
phenytoin	Anticonvulsants
pimozide	Antipsychotics
pindolol	Antihypertensives
Plendil	Antihypertensives
Potiga	Anticonvulsants
prazosin	Antihypertensives
pregabalin	Anticonvulsants
primidone	Anticonvulsants
Pristiq	Antidepressants
Pro-Banthine	Antispasmodics
Prolixin	Antipsychotics
propantheline	Antispasmodics
propranolol	Antihypertensives
protriptyline	Tricyclic Antidepressants
Prozac	Antidepressants
**Q**	
quazepam	Benzodiazepines
quetiapine	Antipsychotics
**R**	
Raudixin	Antihypertensives
Remeron	Antidepressants
reserpine	Antihypertensives
Restoril	Benzodiazepines
Risperdal	Antipsychotics
risperidone	Antipsychotics

Medication	Algorithm
Robaxin	Antispasmodics
Roxidone	Opioids
rufinamide	Anticonvulsants
**S**	
Sabril	Anticonvulsants
Sanctura	Antispasmodics
Sapris	Antipsychotics
Sarafem	Antidepressants
secobarbital	Sedative Hypnotics
Seconal	Sedative Hypnotics
Sectral	Antihypertensives
Serax	Benzodiazepines
Seroquel	Antipsychotics
Serpalan	Antihypertensives
Serpasil	Antihypertensives
sertraline	Antidepressants
Serzone	Antidepressants
Sinequan	Tricyclic Antidepressants
Skelaxin	Antispasmodics
solifenacin	Antispasmodics
Soma	Antispasmodics
Sonata	Benzodiazepines
spironolactone	Antihypertensives
Stelazine	Antipsychotics
Sular	Antihypertensives
Surmontil	Tricyclic Antidepressants
Symbyax	Antipsychotics
**T**	
tapentadol	Opioids
Taztia	Antihypertensives
Tekturna HCT	Antihypertensives
Tegretol	Anticonvulsants
temazepam	Benzodiazepines
Tenex	Antihypertensives
Tenoretic	Antihypertensives
Tenormin	Antihypertensives
terazosin	Antihypertensives
Thalidone	Antihypertensives
thioridazine	Antipsychotics
thiothixene	Antipsychotics

Medication	Algorithm
Thorazine	Antipsychotics
tiagabine	Anticonvulsants
Tiazac	Antihypertensives
timolol	Antihypertensives
tizanidine	Antispasmodics
Tofranil	Tricyclic Antidepressants
tolterodine	Antispasmodics
Topamax	Anticonvulsants
topiramate	Anticonvulsants
Toprol XL	Antihypertensives
torsemide	Antihypertensives
Toviaz	Antispasmodics
Trancot	Sedative Hypnotics
Trandate	Antihypertensives
Tranxene	Benzodiazepines
tranylcypromine	Antidepressants
trazodone	Antidepressants
triamterene	Antihypertensives
triazolam	Benzodiazepines
Tribenzor	Antihypertensives
Tridione	Anticonvulsants
trifluoroperazine	Antipsychotics
Trilafon	Antipsychotics
Trileptal	Anticonvulsants
trimethadione	Anticonvulsants
trimipramine	Tricyclic Antidepressants
trospium	Antispasmodics
Tussionex	Opioids
Tylenol #3	Opioids
**U**	
Urispas	Antispasmodics
**V**	
Valium	Benzodiazepines
valproate	Anticonvulsants
Vasoflex	Antihypertensives
venlafaxine	Antidepressants
verapamil	Antihypertensives
Vesicare	Antispasmodics
Vicodin	Opioids
Vicoprofen	Opioids

Medication	Algorithm
vigabatrin	Anticonvulsants
Viibryd	Antidepressants
vilazodone	Antidepressants
Vimpat	Anticonvulsants
Visken	Antihypertensives
Vivactil	Tricyclic Antidepressants
**W**	
Wellbutrin/Wellbutrin SR	Antidepressants
Wytensin	Antihypertensives
**X**	
Xanax	Benzodiazepines
**Z**	
zaleplon	Benzodiazepines
Zanaflex	Antispasmodics
Zarotin	Anticonvulsants
Zaroxolyn	Antihypertensives
Zayasel	Antihypertensives
Zebeta	Antihypertensives
Ziac	Antihypertensives
ziprasidone	Antipsychotics
Zoloft	Antidepressants
zolpidem	Benzodiazepines
Zonegran	Anticonvulsants
zonisamide	Anticonvulsants
Zyprexa	Antipsychotics

Figure S3: Medication review checklist

FALLS RISK CMR CHECKLIST

Patient: DOB: Date:

FALLS RISK FACTOR(S) IDENTIFIED	FACTOR PRESENT?	NOTES
**FALLS HISTORY**<br>Any falls in the past year?	☐ Yes ☐ No	
Worries about falling?	☐ Yes ☐ No	
Feels unsteady when standing or walking?	☐ Yes ☐ No	
**POSTURAL HYPOTENSION**<br>Patient reported symptoms of lightheadedness or dizziness from lying to standing?	☐ Yes ☐ No	

DRUG CLASSES WITH FALLS RISK	MEDICATION(S) NAME/DOSE/DIRECTIONS	PRESCRIBER
Anticonvulsant agents		
Antidepressant agents		
Antihypertensive agents		
Antipsychotic agents		
Antispasmodic agents		
Benzodiazepines		
Opioids		
Sedative hypnotics		
Tricyclic antidepressants		
Other		

OTHER DRUG THERAPY PROBLEMS (DTPS) MEDICATION(S) NAME/DOSE/DIRECTIONS	PRESCRIBER

STEADI Stopping Elderly Accidents, Deaths & Injuries

UNC ESHELMAN SCHOOL OF PHARMACY

UNC SCHOOL OF MEDICINE

Division of Geriatric Medicine
Center for Aging & Health

Figure S4: Prescriber marketing flyer

STEADI
Stopping Elderly
Accidents, Deaths & Injuries

UNC ESHELMAN SCHOOL OF PHARMACY

UNC SCHOOL OF MEDICINE
Division of Geriatric Medicine
Center for Aging & Health

HELP US PREVENT FALLS IN YOUR OLDER ADULT PATIENTS

What does this mean for your practice?

- This is a pharmacy service supported by a grant from the Centers for Disease Control and Prevention to reduce the risk of falls in older adults through interprofessional collaboration.
- Through collaboration with your community pharmacy, you can meet your quality metrics (e.g. HEDIS measures, annual wellness visits)!
- You may receive a communication from a community pharmacy when they have identified an older adult at increased risk for falling.
- We request you review the pharmacist's recommendations and send your response back to the pharmacy.

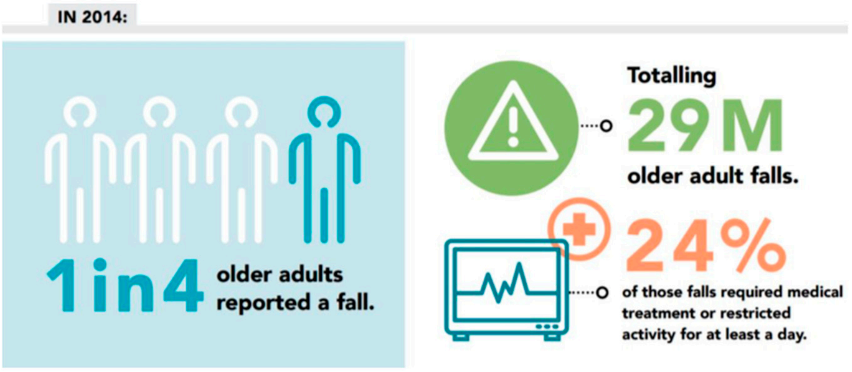

STEADI. Centers for Disease Control and Prevention.

How it works:

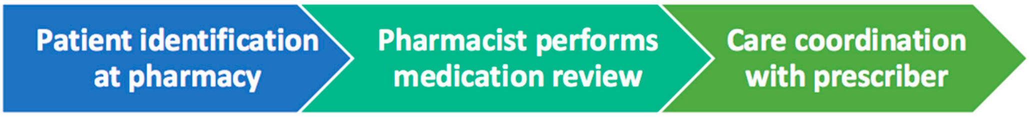

Table S3: North Carolina community-based fall prevention resources

Community-Based Falls Prevention Resources

Eastern NC

- **Healthy Aging NC – A Matter of Balance**
  - Description: a structured, group intervention that utilizes a variety of activities to address physical and cognitive factors affecting fear of falling and to teach fall prevention strategies. Consists of eight 2-hour-long sessions, most including a 30-minute exercise component.
  - Location(s): Williamston, NC
  - Website: http://healthyagingnc.com/workshop/a-matter-of-balance/
  - E-mail: healthyagingncinfo@gmail.com
  - Phone: 828-258-7712

- **Otago Exercise Program**
  - Description: an individualized balance and strength fall prevention program that is delivered by a physical therapist over the course of 52 weeks. Designed to benefit older adults who have sustained falls in the past, have difficulty with gait, balance, or leg strength, and are limited in activities because of concerns of falling.
  - Location(s): Greenville, Kinston, Manteo, Nags Head, Oriental, Rocky Point, Whiteville, Wilmington, Windsor
  - Website: http://healthyagingnc.com/workshop/otago-exercise-program/
  - E-mail: healthyagingncinfo@gmail.com
  - Phone: 828-258-7712

- **Silver Sneakers**
  - A program sponsored by several participating health plans that provides free access to gyms, exercise classes, and other benefits to adults over the age of 65. See website for locations and offerings near you, and to check your patients' eligibility.
  - Location(s): hundreds of locations statewide
  - Website: https://www.silversneakers.com/
  - E-mail: support@silversneakers.com
  - Phone: 866-584-7389

- **More Resources**
  - Healthy Aging NC is looking to be a one-stop shop for evidence-based community-based falls prevention programs. They are continually updating their website to include a complete listing of programs, so check back regularly!
    - Website: http://healthyagingnc.com/falls-prevention-workshops/
    - E-mail: healthyagingncinfo@gmail.com
    - Phone: 828-258-7712

Central NC

- **Healthy Aging NC – A Matter of Balance**
  - Description: a structured, group intervention that utilizes a variety of activities to address physical and cognitive factors affecting fear of falling and to teach fall prevention strategies. Consists of eight 2-hour-long sessions, most including a 30-minute exercise component.
  - Location(s): Creedmoor, Fayetteville, Lexington, Stovall, Wadesboro, Wagram
  - Website: http://healthyagingnc.com/workshop/a-matter-of-balance/
  - E-mail: healthyagingncinfo@gmail.com
  - Phone: 828-258-7712

- **YMCA: Moving for Better Balance**
  - Description: a 12-week evidence-based falls prevention program using the principles and movements of Tai Chi to help older adults increase their strength, improve their balance, and increase their confidence in doing everyday activities.
  - Location(s): Durham, Chapel Hill
  - Website: http://healthyagingnc.com/workshop/ymca-moving-better-balance/
  - E-mail: healthyagingncinfo@gmail.com
  - Phone: 828-258-7712

- **Fit & Strong!**
  - Description: an 8-week multi-component exercise program aimed at older adults with lower extremity osteoarthritis.
  - Location(s): Mayodan, Raleigh, Troy
  - Website: https://www.fitandstrong.org/index.html
  - E-mail: see "Contact Us" form on website
  - Phone: 312-413-9810

- **Otago Exercise Program**
  - Description: an individualized balance and strength fall prevention program that is delivered by a physical therapist over the course of 52 weeks. Designed to benefit older adults who have sustained falls in the past, have difficulty with gait, balance, or leg strength, and are limited in activities because of concerns of falling.
  - Location(s): Asheboro, Benson, Burlington, Buies Creek, Carrboro, Chapel Hill, Durham, Elon, Fuquay-Varina, Greensboro, Hillsborough, Lexington, Morrisville, Pinehurst, Pittsboro, Raeford, Raleigh, Reidsville, Rocky Mount, Roxboro, Sanford, Taylortown, West End, Winston-Salem
  - Website: http://healthyagingnc.com/workshop/otago-exercise-program/
  - E-mail: healthyagingncinfo@gmail.com
  - Phone: 828-258-7712

- **Silver Sneakers**
  - A program sponsored by several participating health plans that provides free access to gyms, exercise classes, and other benefits to adults over the age of 65. See website for locations and offerings near you, and to check your patients' eligibility.
  - Location(s): hundreds of locations statewide
  - Website: https://www.silversneakers.com/
  - E-mail: support@silversneakers.com
  - Phone: 866-584-7389

- **More Resources**
  - Healthy Aging NC is looking to be a one-stop shop for evidence-based community-based falls prevention programs. They are continually updating their website to include a complete listing of programs, so check back regularly!
    - Website: http://healthyagingnc.com/falls-prevention-workshops/
    - E-mail: healthyagingncinfo@gmail.com
    - Phone: 828-258-7712

Western NC

- **Healthy Aging NC – A Matter of Balance**
  - Description: a structured, group intervention that utilizes a variety of activities to address physical and cognitive factors affecting fear of falling and to teach fall prevention strategies. Consists of eight 2-hour-long sessions, most including a 30-minute exercise component.
  - Location(s): Asheville, Black Mountain, Charlotte, Gastonia, Huntersville, Kannapolis, Lincolnton, Marshall, Mooresville, Woodfin
  - Website: http://healthyagingnc.com/workshop/a-matter-of-balance/
  - E-mail: healthyagingncinfo@gmail.com
  - Phone: 828-258-7712

- **YMCA: Moving for Better Balance**
  - Description: a 12-week evidence-based falls prevention program using the principles and movements of Tai Chi to help older adults increase their strength, improve their balance, and increase their confidence in doing everyday activities.
  - Location(s): Asheville, North Wilkesboro
  - Website: http://healthyagingnc.com/workshop/ymca-moving-better-balance/
  - E-mail: healthyagingncinfo@gmail.com
  - Phone: 828-258-7712

- **Fit & Strong!**
  - Description: an 8-week multi-component exercise program aimed at older adults with lower extremity osteoarthritis.
  - Location(s): Charlotte
  - Website: https://www.fitandstrong.org/index.html
  - E-mail: see "Contact Us" form on website
  - Phone: 312-413-9810

- **Otago Exercise Program**
  - Description: an individualized balance and strength fall prevention program that is delivered by a physical therapist over the course of 52 weeks. Designed to benefit older adults who have sustained falls in the past, have difficulty with gait, balance, or leg strength, and are limited in activities because of concerns of falling.
  - Location(s): Belmont, Boone, Charlotte, Columbus, Cullowhee, Franklin, Hendersonville, Hickory, Huntersville, Lenoir, Matthews, Newton, Robbinsville, Salisbury, Valdese, Waynesville
  - Website: http://healthyagingnc.com/workshop/otago-exercise-program/
  - E-mail: healthyagingncinfo@gmail.com
  - Phone: 828-258-7712

Western NC, continued

- **Housing Assistance Corporation Fall Prevention Program**
  - Description: educates low-income elderly and disabled people on the risks and prevention of falling and completes modifications and safety upgrades to their homes so they may remain living independently
  - Location(s): Hendersonville
  - Website: www.housing-assistance.com
  - Phone: 828-692-4744

- **Pleasant Gardens Baptist Church Fall Prevention**
  - Description: Volunteers from the Fishers of Men Sunday School build wheelchair ramps for McDowel County residents.
  - Location(s): Marion
  - Phone: 828-724-4383
  -
- **WNC Fall Prevention Coalition**
  - Description: a listing of resources related to community screening and referrals for falls prevention services in Western North Carolina
  - Website: http://wncfallpreventioncoalition.org/community-screening-referrals/
  - E-mail: use "Contact Us" form on website

- **Silver Sneakers**
  - A program sponsored by several participating health plans that provides free access to gyms, exercise classes, and other benefits to adults over the age of 65. See website for locations and offerings near you, and to check your patients' eligibility.
  - Location(s): hundreds of locations statewide
  - Website: https://www.silversneakers.com/
  - E-mail: support@silversneakers.com
  - Phone: 866-584-7389

- **More Resources**
  - Healthy Aging NC is looking to be a one-stop shop for evidence-based community-based falls prevention programs. They are continually updating their website to include a complete listing of programs, so check back regularly!
    - Website: http://healthyagingnc.com/falls-prevention-workshops/
    - E-mail: healthyagingncinfo@gmail.com
    - Phone: 828-258-7712

Table S4: Project champion survey

Please provide contact information for the project lead at [your pharmacy].
The project lead is the person who was responsible for ensuring that the Falls Risk Study was implemented at your pharmacy.

- ○ First Name ____
- ○ Last Name ____
- ○ Email Address ____

Are you the project lead?

- ○ Yes (1)
- ○ No (2)

What is your role at [your pharmacy]?

- ○ Technician
- ○ Student Intern
- ○ Pharmacist
- ○ Non-Clinical Employee (e.g., office manager)
- ○ Owner
- ○ Other (Please Specify) ____

What education or certifications do you hold? (Check all that apply)

- ☐ Certified Technician (CPhT)
- ☐ Bachelor of Science in Pharmacy (BS Pharm)
- ☐ Doctor of Pharmacy (PharmD)
- ☐ Residency-Trained
- ☐ Board Certification (Please Specify)
- ☐ Clinical Pharmacy Practitioner (CPP)
- ☐ Other (Please Specify)

For how many years have you worked at [your pharmacy]?
If you work at multiple locations within a pharmacy chain, please consider only years worked in the pharmacy location specified above.

- ○ < 1 year
- ○ 1-5 years
- ○ > 5 years

Please rate the usefulness of resources and tools used to conduct a medication review.

	Did Not Use (0)	Not Useful (1)	Somewhat Useful (2)	Very Useful (3)
Medication Checklist	○	○	○	○
High-Risk Medication Index	○	○	○	○
High-Risk Medication Algorithms	○	○	○	○
Patient Education Brochures	○	○	○	○
Community Resources	○	○	○	○
Prescriber Flyer	○	○	○	○
Other (Please Specify)	○	○	○	○

Please rate the usefulness of tools to share recommendations with prescribers.

	Did Not Use (0)	Not Useful (1)	Somewhat Useful (2)	Very Useful (3)
Cover Fax	○	○	○	○
Prescriber Communication Form	○	○	○	○
Prescriber Response Form	○	○	○	○

Please rate the usefulness of the training activities.

	Did not participate (0)	Not Useful (1)	Somewhat Useful (2)	Very Useful (3)
On-boarding Training Webinar	○	○	○	○
NCAP 2017 Workshop	○	○	○	○
Site Visit	○	○	○	○
Coaching	○	○	○	○
APhA CPE Program	○	○	○	○
Quick Tips Emails	○	○	○	○
Quick Tips Webinars	○	○	○	○

Please rate the usefulness of webinar topics.

	Did Not Participate (0)	Not Useful (1)	Somewhat Useful (2)	Very Useful (3)
Workflow integration	○	○	○	○
Engaging with patients	○	○	○	○
Engaging with prescribers	○	○	○	○
Examples from other pharmacies	○	○	○	○
